# EMG Signal Processing for the Study of Localized Muscle Fatigue—Pilot Study to Explore the Applicability of a Novel Method

**DOI:** 10.3390/ijerph192013270

**Published:** 2022-10-14

**Authors:** Sandra B. Rodrigues, Luís Palermo de Faria, António M. Monteiro, José Luís Lima, Tiago M. Barbosa, José A. Duarte

**Affiliations:** 1 FP-I3ID, FP-BHS, Escola Superior de Saúde Fernando Pessoa, Rua Delfim Maia 334, 4200-253 Porto, Portugal; 2 Department of Sports Sciences and Physical Education, Instituto Politécnico de Bragança, 5300-253 Bragança, Portugal; 3 Research Center in Sports, Health and Human Development, 5001-801 Vila Real, Portugal; 4 Research Centre in Digitalization and Intelligent Robotics (CeDRI), Instituto Politécnico de Bragança, Campus de Santa Apolónia, 5300-253 Bragança, Portugal; 5 Laboratório Para a Sustentabilidade e Tecnologia em Regiões de Montanha (SusTEC), Instituto Politécnico de Bragança, Campus de Santa Apolónia, 5300-253 Bragança, Portugal; 6 INESC Technology and Science, 4200-465 Porto, Portugal; 7 CIAFEL, Faculty of Sports, Porto University, Rua Dr. Plácido Costa 91, 4200-450 Porto, Portugal; 8 TOXRUN, University Institute of Health Sciences, Rua Central de Gandra 1317, 4585-116 Gandra, Portugal

**Keywords:** surface electromyography, sEMG, frequency spectrum, fast Fourier transformation, fatigue, signal processing, isokinetic dynamometer

## Abstract

This pilot study aimed to explore a method for characterization of the electromyogram frequency spectrum during a sustained exertion task, performed by the upper limb. Methods: Nine participants underwent an isometric localized muscle fatigue protocol on an isokinetic dynamometer until exhaustion, while monitored with surface electromyography (sEMG) of the shoulder’s external rotators. Firstly, three methods of signal energy analysis based on primer frequency contributors were compared to the energy of the entire spectrum. Secondly, the chosen method of analysis was used to characterize the signal energy at beginning (T1), in the middle (T2) and at the end (T3) of the fatigue protocol and compared to the torque output and the shift in the median frequencies during the trial. Results: There were statistically significant differences between T1 and T3 for signal energy (*p* < 0.007) and for central frequency of the interval (*p* = 0.003). Moreover, the isometric peak torque was also different between T1 and T3 (*p* < 0.001). Overall, there were no differences between the signal energy enclosed in the 40 primer frequency contributors and the analysis of the full spectrum energy; consequently, it was the method of choice. The reported fatigue and the decrease in the produced muscle torque was consistent with fatigue-induced alterations in the electromyogram frequency spectrum. In conclusion, the developed protocol has potential to be considered as an easy-to-use method for EMG-based analysis of isometric muscle exertion until fatigue. Thus, the novelty of the proposed method is to explore, in muscle fatigue, the use of only the main contributors in the frequency domain of the EMG spectrum, avoiding surplus information, that may not represent muscle functioning. However, further studies are needed to investigate the stability of the present findings in a more comprehensive sample.

## 1. Introduction

Sustained exertion associated with physical loads incurred during occupational tasks, namely repetitive high velocity motion, has been associated with a variety of soft tissue disorders [1,2]. Fatigue onset is deemed as a valid measure of injury risk [2,3]. Muscle fatigue is a complex, multifactorial process. The causes of fatigue are classified as (i) central (including brain and spinal cord mechanisms) and (ii) peripheral, neuromuscular, or localized (including peripheral nerve, neuromuscular junction, sarcolemma, excitation-contraction coupling, energy supply, and force generation mechanisms) [4]. Localized muscle fatigue affects motor control, proprioception, and postural stability [5,6,7,8,9,10]. Neuromuscular, peripheral, or localized fatigue is an acute activity-induced reduction in the force/power of a muscle [11]. Thus, an increase in muscle maximum voluntary contraction (MVC) is expected when the muscle is recovering, depending on the oxidative metabolism in working muscles [12]. Moreover, muscle fatigue represents a reduction in the contractile efficiency of the muscle fibers, due to storage of lactic acid, among other mechanisms, that builds up during anaerobic respiration after vigorous exercise.

Fatigue researchers have developed different methodologies to study fatigue based on motion, electroencephalogram, photoplethysmogram, electrocardiogram, galvanic skin response, electromyogram, skin temperature, eye movement, and respiratory data acquired by wearable devices available on the market [13]. Specifically, muscle fatigue is an objective sign of the inability to maintain a certain level of physical effort, which can be studied, for example, by isometric strength test, while muscle biopsy and muscle imaging provide information on the underlining causes [14]. However, these are time-consuming and expensive testing procedures. Conversely, surface electromyography (sEMG) is a feasible alternative and the technique of choice worldwide to study muscle fatigue [14,15].

sEMG is a biosignal depicting the neuromuscular activity that is obtained on the skin surface and can be measured by the amplitude of the signal (e.g., variance, mean-absolute value, root mean square) or its frequency (e.g., Fourier spectrum, mean/median frequency) [16]. sEMG is the sum of the electrical activity of each Motor Unit Action Potentials (MUAPs). Moreover, the shapes and firing rates of MUAPs provide important insights for the diagnosis of neuromuscular disorders [17].

During increasing levels of voluntary contraction, an increase was noted in the muscle conduction velocity (the velocity at which muscle fibers transmit action potentials prior to muscle contraction), mean and median frequency. Moreover, the increase of the initial value of spectral parameters with increasing levels of voluntary contractions is attributed to the recruitment of motor units consisting of progressively larger fibers with progressively higher conduction velocity [18]. Conversely, during constant force sustained isometric contractions, these variables decrease [18], and this phenomenon may be due to fatigue.

It is expected that as the muscles fatigue, marked changes will be detected by the sEMG, namely a slow-down of motor unit action potentials and the synchronization of motor units by the central nervous system. The slowing of motor unit action potentials leads to a decrease of muscle fiber conduction velocity, which is reflected in the EMG frequency domain as a shift of the EMG signal towards lower frequencies [19]. A decrease in the median frequency in the power spectral density has been reported as strongly related to the onset of peripheral muscle fatigue [20,21], although not without criticism, and other methods have also been reported to monitor peripherical fatigue [19,22]; however, these studies looked at only the median frequency, without the contribution of the signal energy, which could represent a bias on the study of muscle biosignal. Additionally, data processing procedures may largely influence analysis and interpretation of EMG spectral characteristics, and so, standardization of methods is needed, and with this work, we intend to develop a novel, standard, and easy to apply method for the study of the signal energy during a local fatigue task. Furthermore, available evidence proposes the use of the entire energy of the EMG spectrum to study the muscle functioning in the frequency domain, including irrelevant EMG information. However, this surplus information may not necessarily represent muscle functioning. Thus, the novelty of the proposed method is to explore the use of only the main contributors in the frequency domain of the EMG spectrum. For this, the following research questions were considered: Which method of energy sampling best represents the total energy of the EMG spectrum? Is the method of choice representative of the fatigue mechanism? Furthermore, the aim of this work was to explore a method for characterization of the electromyogram frequency spectrum, during a sustained exertion upper limb task, which enables the use of only the main contributors in the frequency domain of the EMG spectrum. It is hypothesized that the energy from the main contributors of the power spectral density of the electromyogram will be representative of the full spectrum energy; furthermore, the energy of these main representatives of the spectrum could be used to study the mechanisms of localized muscle fatigue, avoiding surplus spectral information that may not be representative of the true muscle physiology.

## 2. Materials and Methods

### 2.1. Participants

Nine healthy, non-overweight, young volunteer university students (4 females and 5 males), with no history of upper limb musculoskeletal lesions, were recruited for the study. Median (±Interquartile range) characteristics were age 22 ± 3 years; height 168 ± 7 cm; body mass 58.4 ± 14 kg; body mass index (BMI) 20.62 ± 2.39 kg∙m^−2^.

A body mass index cut off value of 24.9 kg m^−2^ was used for the exclusion of overweight participants, since fat tissue acts like a low-pass filter [23]. The protocol also included several clinical tests to address the existence of pathological conditions or pain and ligamentous laxity, since these might influence the outcome [24,25]. Pregnant participants and participants with history of shoulder pathology, shoulder surgery or neurological disorders were also excluded from the study. The present study obtained ethical clearance from the Faculty of Health and Social Science Research Ethics and Governance Committee—ID FREGC-10-035.R1.

### 2.2. Data Collection

The participants were tested in a climate-controlled (21–23 °C) laboratory at the same time of day (±2 h) to minimize the effects of diurnal biological variation. The participants were instructed to arrive at the laboratory in a rested and fully hydrated state, at least 3 h postprandial, and they were asked not to perform any strenuous activity during the day prior to the test.

Height (in m) was measured with the participants standing upright and barefoot against a stadiometer (Seca, Hamburg, Deutschland). Body mass (in kg) was measured by a portable electronic weighting scale (Tanita, Tokyo, Japan). BMI was calculated as the ratio between body mass and body height (kg m**^−^**^2^).

Handedness was assessed using the Dutch Handedness Questionnaire, which contains 16 questions designed to assess hand preference during specific activities. There are three possible answers for each activity, “left hand”, “right hand” and “any of them”. The result is obtained by adding the 16 answers, “left handedness” corresponds to a score of 0, “right handedness” to a score of 2 and “ambidextrous” to a score of 1. The total score can vary from 0 to 32. Participants with a score of 4 or less are “strongly left-handed”, participants with a score of 28 or more are “strongly right-handed” and participants with a score between 5 and 27 are included in the “ambidextrous” group [26].

Participant’s skin over the dominant infraspinatus muscle was cleansed and the Ag/Cl standard electrodes were placed over the muscle belly of the dominant infraspinatus muscle, according to Criswell [27], with a bipolar configuration and an IED of 20 mm. The electromyographic biosignal was measured using a bipolar, 12-bit equipment (Biosignalsplux) at a sampling frequency of 1 kHz, using the acquisition software opensignals (Plux, Lisbon, Portugal).

Participants were placed in a sitting position and securely strapped into the isokinetic chair (System 4 Pro**^®^**, Biodex, New York, NY, USA). Extraneous movement of the upper body was limited by two cross-shoulder harnesses and an abdominal belt. Participants were positioned with 90° of shoulder abduction, 90° of elbow flexion and 0° of shoulder rotation, as previously reported [28,29,30,31,32,33,34]. The choice of the scapular plane enables a more functional approach of the movement, and the literature supports the choice of 40° anterior to the coronal or frontal plane [35,36,37]. The joint angles were measured using a universal baseline plastic goniometer.

The warm-up consisted of five submaximal isometric contractions of 5 s, followed by 3 min of rest. The participants were then asked to perform one maximal isometric voluntary contraction (MIVC) of the shoulder external rotators until exhaustion while a strong verbal encouragement was provided by the researcher. The experimental protocol was consistent with the process of carrying out training until muscle failure, defined as the inability to sustain an isometric contraction while maintaining proper form. This criterion was enhanced by verbal report of exhaustion. Reaching the end, the participants perception of exertion was assessed using the Borg Scale. The Borg Rating of Perceived Exertion (RPE) is based on the physical sensations a person experiences during physical activity, including increased heart rate, increased respiration or breathing rate, increased sweating and muscle fatigue. Although this is a subjective measure, the exertion rating, based on a 6 to 20 rating scale, may provide an adequate estimate of the actual heart rate during physical activity [38].

### 2.3. Data Processing

The corresponding EMG time frames on the isokinetic data were used for peak torque analysis of three moments (1 s at the beginning, 1 s in the middle and 1 s in the end) of the exhaustive protocol. EMG processing was computed on MATLAB**^®^** R2022A. The signal was initially filtered with a Notch Filter of 50 Hz, due to the DC power line. Afterward, it was detrended and transformed into Volts.

**Figure 1:**
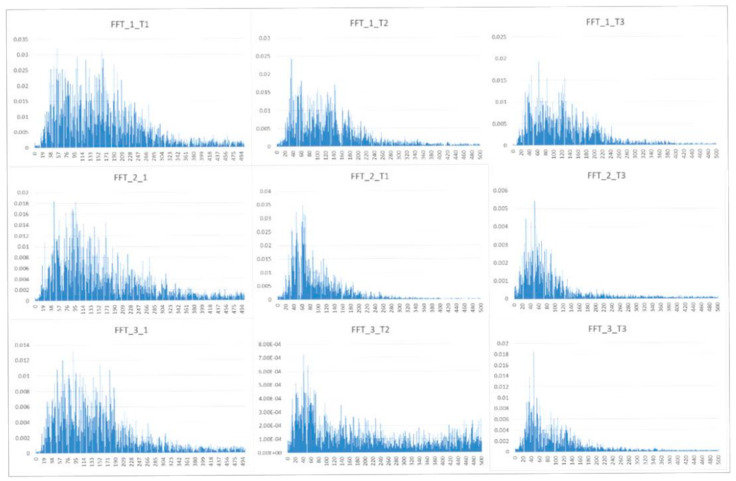
Examples of fast Fourier transformation (FFT) analysis of three participants in different time frames (in the beginning, T1, in the middle, T2, and in the end of the trial, T3), displayed as a plot of frequency in Hz (*x*-axis) against signal energy (*y*-axis).

The entire frequency domain was computed via Fast Fourier Transformation (FFT). The FFT transformed the time domain signal into a frequency domain representation of that signal, and consequently, it was created a description of the distribution of the energy in the signal as a function of frequency. This was displayed as a plot of frequency (*x*-axis) against energy (*y*-axis) called a power spectral density graph (Figure 1).

The FFT spectrum was then further evaluated choosing the 10, 20, 30 and 40 primer frequency contributors, representing the main energy at each of the specified groups of frequencies in the spectrum.

Each of the samples (10, 20, 30 and 40 primer frequency contributors) were compared to the energy of the entire frequency spectrum.

To evaluate the signal energy, the following equation was computed [39,40]:

(1) $$\sum \frac{1}{2}\;A_{k\;}^{2}∆t$$

where *A_k_* represents the frequency amplitude of a given frequency and ∆*t* the time frame of analysis, (1 s). Equation (1) was computed for each participant.

The central frequency of each interval (10, 20, 30 and 40 primer frequency contributors—abbreviated to P) was computed using the expression:

(2) $$\frac{\min freq+\max freq}{2}\;$$

where “min *freq*” represents the minimum frequency in the given interval and “max *freq*” represents the maximum value of frequency in the given interval.

The chosen method of analysis was used to characterize the signal energy and the central frequency at the beginning (T1—first second), in the middle (T2—one second in the middle of the signal) and at the end (T3—last second) of the exhaustive protocol.

### 2.4. Statistical Analysis

The data were tested for distribution using the Shapiro–Wilk test and were found to be not normally distributed. Consequently, the non-parametric test of Friedman’s two-way analysis of variance by ranks was employed to compare between the different type of analysis based on the prime frequency contributors of the EMG density spectrum; furthermore, it was also used to compare the variables between moments. Pairwise comparisons were performed, with a Bonferroni correction for multiple comparisons. For consistency, the median value and interquartile range was reported. Effect sizes were calculated based on Kendall W statistics for Friedman’s test [41]. A priori level of significance was set at *p* < 0.05 for all comparisons. The data were analyzed using both the Microsoft^®^ Excel^®^ for Microsoft 365 (v. 2206) and IBM SPSS Statistics 28.0.

## 3. Results

Table 1 shows the median and interquartile range for the results of the Borg scale and the time until reported fatigue. Data analysis suggests an apparent interindividual homogeneity of both time and demand, in terms of perceived exertion, of the protocol.

To test which of the primer frequency contributors would better represent the energy of the full spectrum, the median of the energy of each of the four methods were compared to the full spectrum (Table 2). Table 2 compares the signal energy using four methods (10P, 20P, 30P or 40P) of primer frequency contributors and the signal energy of the full spectrum. Signal energy was statistically significantly different between the different methods of processing χ^2^(4) = 108.00, *p* < 0.001, effect size of 1.00. According to the pairwise comparison, the only representative that was not different from the full spectrum was the 40 primer frequency contributors. Corresponding to this finding, all the subsequent analyses will take into consideration the 40 primer frequency contributors’ method.

**Figure 2:**
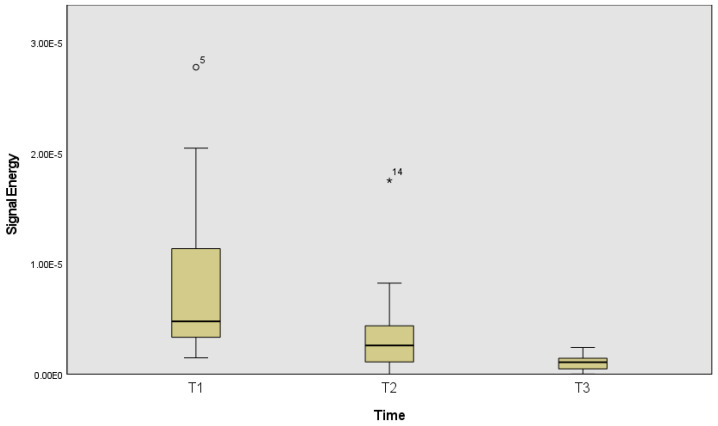
Signal energy at the beginning of acquisition (T1), in the middle (T2) and during the reported fatigue (T3). The circle represent a value above the 3rd quartile + 1.5xinterquartile range, while the star represent the 3rd quartile + 3xinterquartile range.

**Figure 3:**
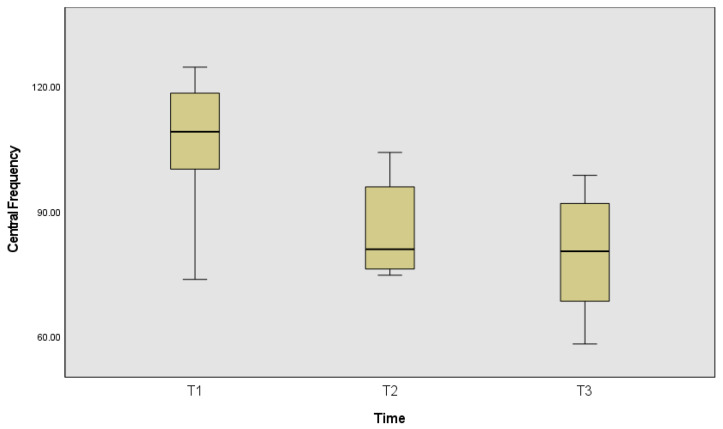
Central frequencies in Hz, at the beginning of acquisition (T1), in the middle (T2) and during the reported fatigue (T3).

The following figures represent the signal energy across time (Figure 2) and central frequency across time (Figure 3).

Signal energy was statistically significantly different across the different time points (T1, T2 and T3) χ^2^_(2)_ = 9.56, *p* = 0.008, effect size of 0.53. Pairwise comparisons were performed, with a Bonferroni correction for multiple comparisons. Pairwise comparison only identified differences between the first and the last (*p* = 0.007) time frame (beginning and ending of the fatigue protocol).

The computed central frequency is also statistically significantly different between time frames χ^2^_(2)_ = 10.75, *p* = 0.005, effect size of 0.67; thus, differences in the pairwise comparison only identified differences between the first and the last time frame (*p* = 0.003).

**Figure 4:**
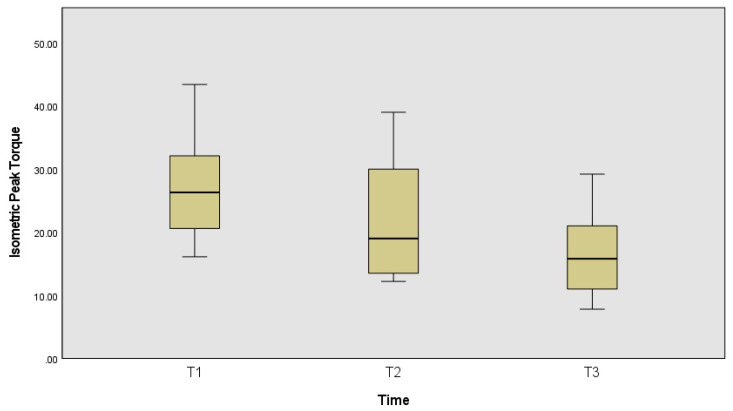
Isometric peak torque, in N.m, at the beginning of the measurement protocol (T1), in the middle (T2) and during the reported fatigue (T3).

The isometric peak torque (Figure 4) was also statistically significantly different between time frames χ^2^_(2)_ = 16.22, *p* < 0.001, effect size of 0.90, with significant statistical differences between the first and the last time frame (*p* < 0.001).

## 4. Discussion

The present study explored a method for characterization of the electromyogram frequency spectrum, during a sustained exertion upper limb task, which enables the use of only the main contributors in the frequency domain of the sEMG spectrum.

Based on the classical document of Enoka and Duchateau [42], the authors put in perspective the use of the two-domain fatigue concept, in which fatigue is defined as a disabling symptom where physical and cognitive function is limited by interactions between performance fatigability and perceived fatigability, and as a symptom, fatigue could only be measured by self-report, as in the present study, and quantified either as a trait characteristic or a state variable. Furthermore, the authors claim that because of such a definition, the word fatigue should not be preceded by an adjective (e.g., central, mental, muscle, peripheral and supraspinal) to suggest the locus of the changes responsible for an observed level of fatigue. Rather, mechanistic studies should be performed with validated experimental models to identify the changes responsible for the reported fatigue. In this sense, one validated method of fatigue analysis is the surface electromyogram, which represents the recording of the electrical activity of the muscle and is the interferential sum of tissue-filtered motor unit action potentials, and embodies a pattern characterizing the general state of the muscle examined [43]. Moreover, peripheral fatigue, which in the classical sense is the type of fatigue under investigation, may occur either at the neuromuscular junction and cell membrane (excitation), the calcium release mechanism (activation) or at the sliding filaments (contractile processes) [44]. A progressive spectral compression of the EMG signal towards lower frequencies has been suggested to be related to peripheral fatigue during sustained contractions [45]. Thus, muscle fatigue has traditionally been studied by means of the mean power frequency (MPF) [21,44] and the results from the present study seem to show the same data tendency. Furthermore, while the change in the frequency domain of the electromyogram has been explored in the literature, during fatigue, it is important to understand the changes at spectral energy level and use only the representative energy of the acting muscle, avoiding surplus information that may not be representative of the true muscle signal.

In terms of general description of the results, the task was classified by the participants as hard [38] and the participants were able to maintain the effort for 75.28 s. Besides studying the mean power frequency, which showed a tendency towards lower frequencies, with statistically significant differences between the beginning and the end of the protocol, the present study also analyzed the spectral energy during the fatiguing protocol. For this, a method of study was developed to better understand the mechanism of fatigue at the spectral level of energy. Since there was no statistically significant difference between the signal energy of the 40 primer frequency contributors of the signal and the full energy spectrum, the energy of the 40 primers contributors was chosen to study the muscular signal energy using the spectral analysis of the electromyogram.

Furthermore, the method of reported fatigue induced statistically significant differences in the signal’s energy, the central frequency, and the peak torque, namely between the beginning of the acquisition and the timeframe of reported fatigue, suggesting that the protocol of isometric fatigue induced a statistically significant reduction not only at the level of the spectral energy, but also at the level of torque production. This peripheric or neuromuscular fatigue can under certain conditions be reflected in a decreased performance, and many investigators use this as a definition for fatigue [46], and the present results also show this data tendency toward lower force production with fatigue. Furthermore, this decline in maximal voluntary contraction (MVC) is a classic index of muscle fatigue [42].

As suggested in the taxonomy proposed by Kluger and Krupp [47], the concept of fatigue should acknowledge its two attributes: (1) performance fatigability—the decline in an objective measure of performance over a discrete period of time; (2) perceived fatigability—changes in the sensations that regulate the integrity of the performer. The present study took these concepts into consideration, aiming to identify the ability of the proposed method to detect muscular changes associated with fatiguing mechanisms.

However, spectral analyses of EMG data may provide a more in-depth assessment of changes in muscle dynamics occurring across different exercise intensities than more traditional, amplitude-derived methods; while somewhat controversial, results derived from spectral analysis have been associated with a variety of factors including fiber type (i.e., Type I vs. II), conduction velocity and muscular fatigue [48]. Lately, the published literature has a tendency towards using the wavelet transformation of the EMG data [48,49], which converts a signal from the time-domain to the time-frequency domain, such that for each point in time, there is a decomposition of the signal around that point in time into its constituent frequencies. Nevertheless, the spectral signal energy is not under consideration, and for this, the present study tries to overcome this limitation in the available literature.

The application of the present protocol to a sample of normal subjects allowed the analysis on the applicability of the method, and the results were consistent with the literature. Nevertheless, this is a preliminary pilot study that analyzed the applicability of the present method in a limited sample of only nine subjects. While taking into account the changes not only in the EMG signal, namely in the energy of the power spectral density and the shift toward lower frequencies, the present study also included a torque output, to understand the implications of such a change in the function of the muscle. However, it is acknowledged that further analysis of the present findings could add to the present evidence, namely including other methods of analysis such as the Bland and Altman statistics for the agreement between methods analysis and intraclass correlations reporting. Nevertheless, this analysis should be performed in future studies with larger samples.

## 5. Conclusions

Overall, there were no differences between the signal energy enclosed in the 40 primer frequency contributors and the analysis of the full spectrum energy. Consequently, the 40 primer frequency contributors’ method for the study of EMG signal energy was the method of choice to study the mechanism of local fatigue in the upper limb task. The reported fatigue and the decrease in the produced muscle torque was consistent with fatigue-induced alterations in the electromyogram frequency spectrum and spectral signal energy. In conclusion, the developed protocol has potential to be considered as an easy-to-use method for EMG-based analysis of isometric muscle exertion until fatigue; however, the limited number of subjects included in the present study induces the need to further investigate the stability of the present findings in a more comprehensive and variable sample of healthy subjects.

## Figures and Tables

**Table 1:** Median and interquartile range (IR) values of the time until reported fatigue and the Borg scale of perceived exertion.

	Median	IR
Borg Scale	15.00	2.00
Time until reported fatigue [s]	75.28	7.55

**Table 2:** Comparison of the signal energy using four methods (10P, 20P, 30P or 40P) of primer frequency contributors and the signal energy of the full spectrum.

Method of Analysis	Median	Interquartile Range
10P energy	1.06 × 10^−6^	1.52 × 10^−6^
20P energy	1.63 × 10^−6^	2.46 × 10^−6^
30P energy	2.07 × 10^−6^	3.17 × 10^−6^
40P energy	2.34 × 10^−6^	3.69 × 10^−6^
Full spectrum energy	3.5 × 10^−6^	6.7 × 10^−6^
	*p* < 0.001 *	

* Represents significance.
